# Cloning a Chymotrypsin-Like 1 (CTRL-1) Protease cDNA from the Jellyfish *Nemopilema nomurai*

**DOI:** 10.3390/toxins8070205

**Published:** 2016-07-05

**Authors:** Yunwi Heo, Young Chul Kwon, Seong Kyeong Bae, Duhyeon Hwang, Hye Ryeon Yang, Indu Choudhary, Hyunkyoung Lee, Seungshic Yum, Kyoungsoon Shin, Won Duk Yoon, Changkeun Kang, Euikyung Kim

**Affiliations:** 1 Department of Pharmacology and Toxicology, College of Veterinary Medicine, Gyeongsang National University, Jinju 660-701, Korea; yunwi0510@naver.com (Y.H.); skyward2@hanmail.net (Y.C.K.); hotjjang002@naver.com (S.K.B.); pooh9922@hanmail.net (D.H.); tkwk565@naver.com (H.R.Y.); induchoudhary2u@gmail.com (I.C.); leehy@gnu.ac.kr (H.L.); ckkang@gnu.ac.kr (C.K.); 2 Marine Environment Research Center, Korea Institute of Ocean Science and Technology (KIOST), Geoje 656-834, Korea; syum@kiost.ac.kr; 3 Faculty of Marine Environmental Science, University of Science and Technology (UST), Geoje 656-834, Korea; 4 Ballast Water Research Center, Korea Institute of Ocean Science and Technology (KIOST), Geoje 656-834, Korea; ksshin@kiost.ac.kr; 5 Headquarters for Marine Environment, National Fisheries Research & Development Institute, Shiran-ri, Gijang-eup, Gijang-gun, Busan 619-705, Korea; wdyoon@korea.kr; 6 Institutes of Agriculture and Life Science, Gyeongsang National University, Jinju 660-701, Korea; 7 Engineering Research Institute, Gyeongsang National University, Jinju-si 660-701, Korea

**Keywords:** *Nemopilema nomurai*, amidolytic kinetic assay, cloning a chymotrypsin-like 1 (CTRL-1) protease, full-length cDNA sequence, genomic DNA sequence

## Abstract

An enzyme in a nematocyst extract of the *Nemopilema nomurai* jellyfish, caught off the coast of the Republic of Korea, catalyzed the cleavage of chymotrypsin substrate in an amidolytic kinetic assay, and this activity was inhibited by the serine protease inhibitor, phenylmethanesulfonyl fluoride. We isolated the full-length cDNA sequence of this enzyme, which contains 850 nucleotides, with an open reading frame of 801 encoding 266 amino acids. A blast analysis of the deduced amino acid sequence showed 41% identity with human chymotrypsin-like (CTRL) and the CTRL-1 precursor. Therefore, we designated this enzyme *N. nomurai* CTRL-1. The primary structure of *N. nomurai* CTRL-1 includes a leader peptide and a highly conserved catalytic triad of His^69^, Asp^117^, and Ser^216^. The disulfide bonds of chymotrypsin and the substrate-binding sites are highly conserved compared with the CTRLs of other species, including mammalian species. *Nemopilema nomurai* CTRL-1 is evolutionarily more closely related to Actinopterygii than to Scyphozoan (*Aurelia aurita*) or Hydrozoan (*Hydra vulgaris*). The *N. nomurai* *CTRL1* was amplified from the genomic DNA with PCR using specific primers designed based on the full-length cDNA, and then sequenced. The *N. nomurai* *CTRL1* gene contains 2434 nucleotides and four distinct exons. The 5′ donor splice (GT) and 3′ acceptor splice sequences (AG) are wholly conserved. This is the first report of the *CTRL1* gene and cDNA structures in the jellyfish *N. nomurai*.

## 1. Introduction

Serine proteases are enzymes that hydrolyze specific peptide bonds in proteins via an activated serine residue in their substrate-binding sites [1]. They have a number of physiological and pathological roles in mammals, such as digestion, immune response, cellular differentiation, and thrombosis [2,3]. Based on substrate specificity, they can be categorized as either trypsin-like, elastase-like or chymotrypsin-like enzymes [4]. The chymotrypsin-like proteases cleave peptides bonds on the carboxyl side of phenylalanine, tyrosine, or tryptophan residues [5]. In mammals, many physiological processes are regulated by chymotrypsin-like proteases, including apoptosis, signal transduction [6,7], reproduction [8], hemostasis, and immune responses [9]. Therefore, several research groups have purified and characterized chymotrypsin-like proteases from various organisms. For example, the chymotrypsin-like protease from *Bacillus amyloliquefaciens* FCF-11 shows potential application as a thrombolytic agent [10], and a new chymotrypsin-like serine protease, involved in dietary protein digestion has been purified from *Scorpio maurus*, followed by the characterization of its biochemical properties [11]. In marine animals, Xiu et al. [12] purified a chymotrypsin-like protease from the Chinese shrimp, *Fenneropenaeus chinensis*, and demonstrated its inhibitory effect on cell adhesion for innate immunity. *Rhopilema nomadica,* a phylum Cnidaria is known to express chymotrypsin enzyme [13]. Interestingly, there are several serine proteases that have been characterized as toxins in the venoms of poisonous animals, including snakes, bees, etc. In snake venom, they can inhibit blood coagulation in victims and spread toxic components throughout the bloodstream [14]. In the case of bee venom, serine protease components are well known to play as allergens [15].

A decade ago, *N. nomurai*, one of the largest cnidarian jellyfish in the world, began to bloom in East Asian marginal seas, such as the Bohai Sea, Yellow Sea, China Sea, and East Sea [16]. Unusual blooms of this jellyfish have seriously damaged local fisheries almost every year since 2002 [16] and its envenomation of humans has increased since 1983. In general, the venom of *N. nomurai* shows various types of toxicities, including hemolytic [17], hepatotoxic [18] and cardiotoxic [19] responses, and it may have caused fatalities [20]. Therefore, the biological roles of the proteins in *N. nomurai* jellyfish venom must be investigated to more comprehensively understand the biology of *N. nomurai*. Several groups have focused on verifying the genomic sequence of *N. nomurai*, but to date, only the whole mitochondrial genomic sequences have been published for several jellyfish, including their telomeres, including the scyphozoa *Aurelia aurita* [21], the hydrozoa *Hydra oligactis* [22], *Hydra magnipapillata* [23] and the cubozoa *Alatina moseri* [24]. Despite these efforts, only a few cDNA sequences of *N. nomurai* have been reported, including that of lectin [25].

In this study, we cloned the genomic and cDNA sequences of a chymotrypsin-like proteinase 1 (CTRL-1) from the jellyfish *N. nomurai*, collected off the coast of Korea.

## 2. Results

### 2.1. Specific Substrate of Crude N. nomurai Nematocyst Extract

**Figure 1:**
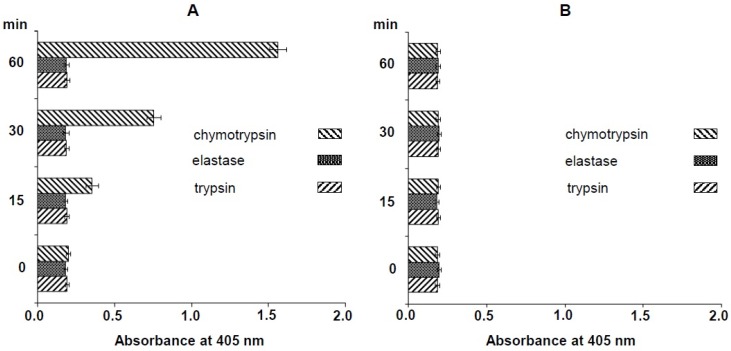
Amidolytic activation (**A**) and inhibition assay (**B**) of *N. nomurai* nematocyst extract using several serine protease substrates. phenylmethanesulfonyl fluoride (PMSF) was used as the serine protease inhibitor.

The nematocyst extract of *N. nomurai* was assayed for amidolytic activity using several substrates. Only chymotrypsin substrate was cleaved specifically and this activity was inhibited by phenylmethanesulfonyl fluoride (PMSF). Neither the elastase nor the trypsin substrate was cleaved (Figure 1).

### 2.2. N. nomurai CTRL-1 cDNA Cloning and Sequence Analysis

**Figure 2:**
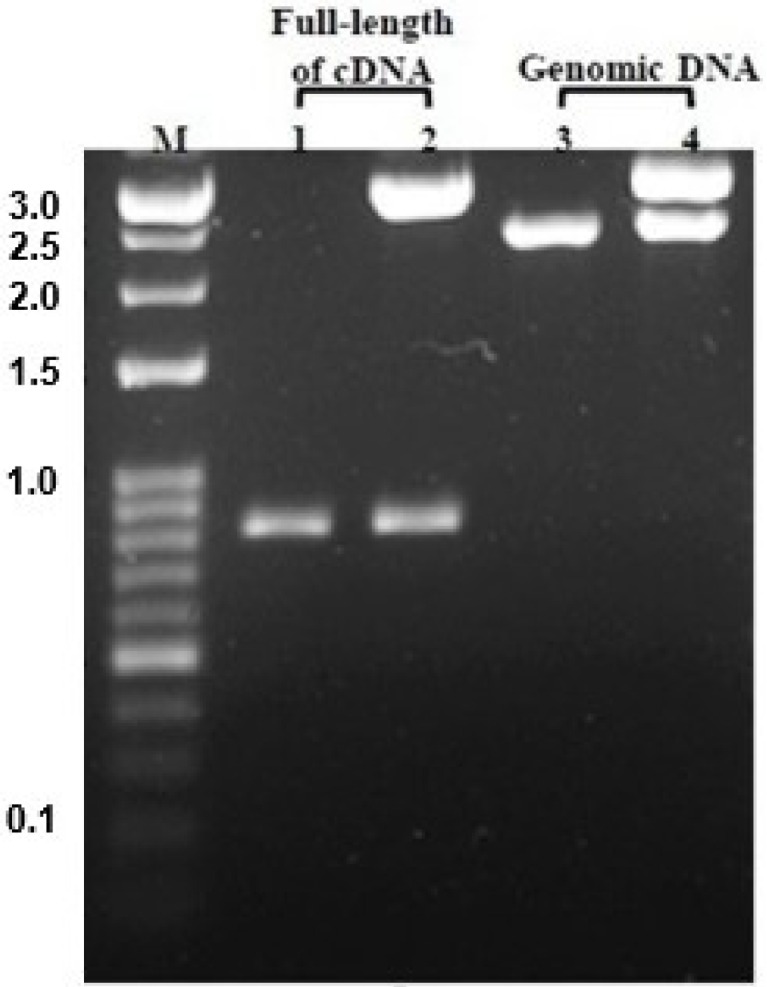
Agarose gel electrophoresis (1.2%) of the pGEM-T/CTRL-1 cDNA and pGEM-T/CTRL-1 genomic DNA after *EcoR*I digestion. M: 100-bp size marker; lane 1: full-length CTRL-1 cDNA PCR product; lane 2: pGEM-T/CTRL-1 cDNA; lane 3: CTRL-1 genomic DNA PCR product; lane 4: pGEM-T/CTRL-1 genomic DNA.

**Figure 3:**
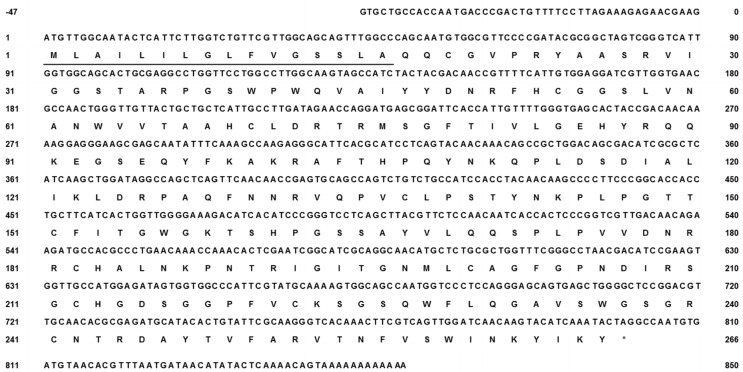
Full-length cDNA and deduced amino acid sequence of *N. nomurai* chymotrypsin-like protease (CTRL-1). The asterisk and single underline indicate the in-frame stop codon (TAG) and the predicted signal peptide (SignalP 4.1 server), respectively. The double underline indicates the polyadenylation signal (TTTAAT), * represents “Stop”.

**Figure 4:**
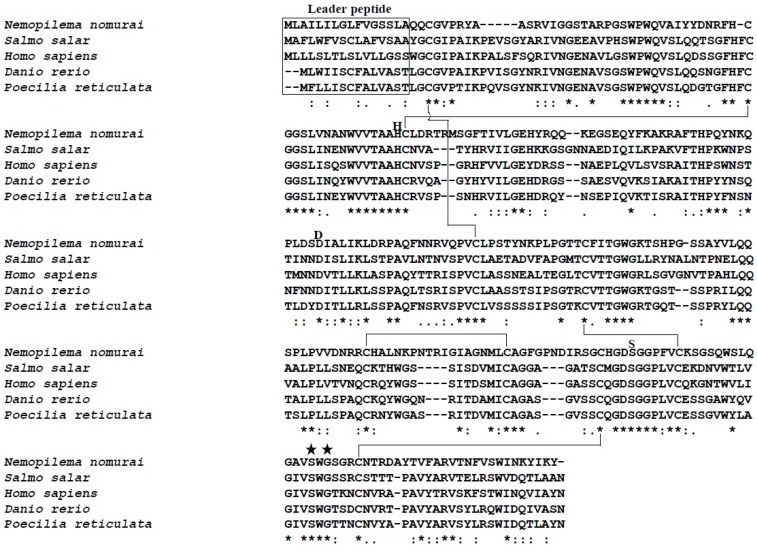
Alignment of the protein sequences of four chymotrypsin-like proteases with the deduced sequence of *N. nomurai* CTRL-1. The leader peptides of the chymotrypsins are indicated. The *lines* between the conserved cysteines indicate the actual disulfide bonds found in the chymotrypsins. The letters H, D, and S indicate the positions of the active-site residues His^69^, Asp^117^, and Ser^216^, respectively. The star mark (★) indicates the substrate-binding site. Identical, similar, and weakly similar amino acids are indicated by asterisks, colons, and dots, respectively. Gaps are indicated by dashes.

The cDNA library of the *N. nomurai* CTRL-1 gene was constructed to identify the full-length cDNA sequence (GenBank accession no. KU668696) using total RNA that was extracted from the tentacle. The PCR product of full-length cDNA (Figure 2, lane 1) was cloned into the pGEM-T Easy vector and the clone was confirmed with *EcoR*I digestion on 1.2% agarose gel electrophoresis (Figure 2, lane 2). The CTRL-1 cDNA size is 850 bp, including an 801-bp open reading frame (ORF) that encodes 266 amino acids (Figure 3). The deduced amino acid sequence has a putative signal peptide between residues 17 and 18 and the mature polypeptide has 249 residues. The cDNA sequence of CTRL-1 contains an in-frame stop codon (TAG) and predicted polyadenylation signal (TTTAAT) (Figure 3). A blast analysis of the sequenced CTRL-1 cDNA showed a high degree of identity with those Scyphozoa and Hydrozoa and Actinopterygii. (Table 1). An alignment of the deduced amino acid sequences of *N. nomurai* CTRL-1 and CTRL-1 of another four species (*Salmo salar*, *Danio rerio*, *Poecilia reticulata*, and *Homo sapiens*) showed that the 10 cysteine residues involved in disulfide bonds in chymotrypsin, and the catalytic triad residues (His^69^, Asp^117^, and Ser^216^) are highly conserved. The substrate-binding sites are also highly conserved (Figure 4).

### 2.3. Evolutionary Relationships of the N. nomurai CTRL-1 Gene

**Figure 5:**
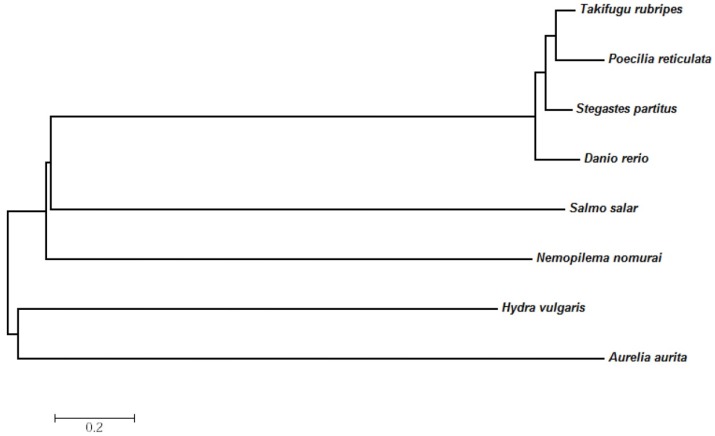
The phylogenetic tree of the *N. nomurai* *CTRL-1* gene, constructed with the MEGA ver. 6.06 software (Center for Evolutionary Medicine and Informatics, Arizona state University, Tempe, AZ, USA), using the neighbor-joining method, Poisson model, and uniform rates. The sequence accession numbers are *Hydra vulgaris* (XP_002164641.1), *Aurelia aurita* (AAO12213.1), *Danio rerio* (NP_001004582.1), *Salmo salar* (NP_001134565.1), *Takifugu rubripes* (XP_003966055.1), *Poecilia reticulata* (XP_008430618.1), and *Python bivittatus* (XP_007421153.1).

To determine the evolutionary relationships of the *N. nomurai* CTRL-1 protein with those of other groups, a phylogenetic tree was constructed using the neighbor-joining method. *N. nomurai* CTRL-1 was more closely related to the Actinopterygian protein than to the Scyphozoan and Hydrozoa proteins. Within the Cnidarian, the *N. nomurai CTRL-1* gene was evolutionarily more closely related to the *H. vulgaris* gene than to the *A. aurita* gene (Figure 5).

### 2.4. Genomic Structure of N. nomurai CTRL-1

**Figure 6:**
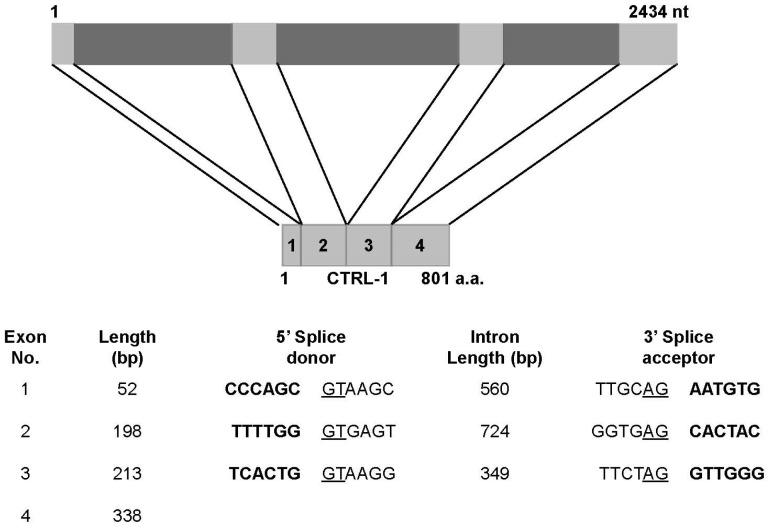
The organization of the *N. nomurai* *CTRL-1* gene. Upper panel shows that the *CTRL-1* gene contains four distinct exons. Lower panel shows the exon and intron organization. The 5′ acceptors and 3′ donor splice sites are underlined. Bold print indicates exons.

The *N. nomurai* *CTRL-1* gene (GenBank accession no. KU668697) was amplified with PCR from the genomic DNA with specifically designed primers based on the full-length cDNA sequence. The PCR product (Figure 2, lane 3) was cloned into the pGEM-T Easy vector and the clone was confirmed with *EcoR*I digestion on 1.2% agarose gel electrophoresis (Figure 2, lane 4). Analysis of the *N. nomurai* *CTRL-1* gene structure showed that *CTRL-1* contains four distinct exons, with length ranging from 52 to 338 bp. Both the canonical 5′ donor and 3′ acceptor splice sites are present in each intron (Figure 6 and Supplementary Figure S1).

## 3. Discussion

To better understand the physiological and pathological features of the *N. nomurai* sting, the venom proteins must be identified. However, there are several challenges in studying *N. nomurai* venom proteins because there is no standard, powerful method for the purification of venom proteins. Similarly, no information is available on the genome, transcriptome, or proteome of *N. nomurai*. For these reasons, we used an amidolytic activity assay to preliminarily identify the proteins present in *N. nomurai* venom. Several substrates, including matrix metalloproteinases and serine proteases, were degraded and changed color in the amidolytic activity assay. Interestingly, only chymotrypsin substrate, a serine protease, was cleaved, whereas the other serine proteases, elastase and trypsin, were not. To confirm these results, the crude venom extract was treated directly with PMSF, a serine protease inhibitor, and this activity was abolished (Figure 1). The present results indicate that *N. nomurai* jellyfish has a chymotrypsin protease activity. As a serine protease, chymotrypsin has an antiinflammatory activity, especially by inducing the breakdown of fibrin clot in a process called fibrinolysis [26,27]. Interestingly, many snake venoms are well known to contain fibrinogenolytic and fibrinolytic enzymes [28] for spreading the venom quickly and efficiently in prey’s lymphatic system to subdue. At the same time, the serine proteinases in snake venoms are also considered as digestive enzymes to absorb the nutrients needed [29]. Like those in snake venoms, the chymotrypsin in jellyfish venom has antithrombotic activity (unpublished data from our laboratory, in preparation for publication). Therefore, these results suggest that jellyfish chymotrypsin may have roles as a venom component as well as a digestive enzyme.

To identify the chymotrypsin protease gene of *N. nomurai*, we synthesized the first-strand cDNA from the total RNA extracted from tentacles, using a primer designed from partial transcriptome sequence data or oligo(dT)_18_. RACE PCRs were used to obtain the full-length cDNA sequence of the *N. nomurai* chymotrypsin protease. The CTRL-1 cDNA sequence of *N. nomurai* has 850 nucleotides, with a polyadenylation signal (TTTAAT), and is shown in Figure 3. The complete ORF contains 801 nucleotides encoding 266 amino acids. A BLAST analysis showed that the *N. nomurai* chymotrypsin protease shares high identity with chymotrypsin-like 1 (CTRL-1) of many animal classes, including mammals (Table 1). Therefore, *N. nomurai* has CTRL-1 protease activity. The primary structure of the protein was predicted with InterProscan (EMBL-EBI, Hinxton, UK), and the *N. nomurai* CTRL-1 protein contains several conserved domains, including the peptidase S1A, chymotrypsin-type domain found in coagulation factor XII, the complement B/C2 domain, and the haptoglobin domain, which are found in other CTRL-1 proteins (data not shown). The SignalP 4.1 tool (Center for Biological Sequnece analysis, Department of systems Biology Techbical University of Denmark, Lyngby, Denmark) predicted that *N. nomurai* CTRL-1 contains the signal peptide, MLAILILGLFVGSSLA. The motif of substrate-binding site and its catalytic triad sites are also highly conserved (Figure 4).

To determine the evolutionary relationships between *N. nomurai* CTRL-1 and other CTRL-1 proteins, a phylogenetic tree was constructed with the MEGA program (version 6.06), which showed that *N. nomurai* CTRL-1 is slightly closer to actinopterygian CTL-1 than to the proteins of other classes. However, when the gene was compared among other cnidarians, it was clearly more closely related to the *A. aurita* gene than to the *H. vulgaris* gene (Figure 5).

The *N. nomurai* *CTRL1* gene was amplified from genomic DNA with PCR. It contains 2434 nucleotides and has four distinct exons (Figure 6). Interestingly, the conserved dinucleotide sequences at the 5′ donor splice site (GT) and the 3′ acceptor splice site (AG) in the introns [30,31] are highly conserved (Supplementary Figure S1).

In this study, we have for the first time demonstrated CTRL-1 enzymatic activity from *N. nomurai* jellyfish venom. Moreover, we have determined its full-length cDNA and gene sequence. Although we do not presently have enough evidence to prove *N. nomurai* CTRL-1 as a toxin, there are several serine proteases that have been previously identified as toxins in other venomous animals. So far, only a few toxin genes have been proposed in the Cnidarian species, especially for jellyfish and their mechanism of actions have been hardly understood yet. To clarify the physiological or toxinological features of CTRL-1, it is required to have further works on its functional roles with the isolation of venom-derived protein or its recombinant product in the near future.

## 4. Experimental Procedures

### 4.1. Materials

*Nemopilema nomurai* jellyfish were collected from around the coast of the Republic of Korea and immediately transferred to the laboratory on ice. The nematocysts were prepared for an analysis of enzyme activity and the tentacles for mRNA and genomic sequencing.

### 4.2. Nematocyst Isolation and Venom Extraction

The amidolytic kinetic assay was used to identify the proteins in the nematocysts. For this experiment, the tentacles were dissected and their nematocysts isolated. Briefly, the dissected tentacles were autolysed in seawater overnight. The precipitate was centrifuged at 1000× *g* (4 °C) for 5 min. The pellet was lyophilized and stored at −20 °C. The venom was extracted from the freeze-dried nematocysts. Briefly, the venom was extracted from 50 mg of nematocysts using glass beads (approximately 8000 beads; 0.5 mm in diameter) and 1 mL of ice-cold (4 °C) phosphate-buffered saline (pH 7.4). The samples were shaken in a mini bead mill at 3000 rpm five times for 30 s each, with intermittent cooling on ice. The venom extracts were then transferred to a new microcentrifuge tube and centrifuged at 15,000× *g* (4 °C) for 30 min. The isolated supernatant was then centrifuged at 15,000 rpm (4 °C) and used for the amidolytic kinetic assay. The protein concentration in the venom was determined with the Bradford method (Bio-Rad, Hercules, CA, USA) and the venom was used based on its protein concentration.

### 4.3. Amidolytic Activity Assay of N. nomurai Venom

*N*-Succinyl-Ala-Ala-Ala-*p*-nitroanilide, *N*-α-benzoyl-dl-Arg-nitroanilide, and *N*-succinyl-Ala-Ala-Pro-Phe-*p*-nitroanilide were used as the substrates to test for elastase, trypsin, and chymotrypsin activities, respectively. The substrates (0.5 mM) were mixed with 0.1 mg of isolated nematocysts for 1 h at 37 °C and the change in absorbance at 405 nm was recorded (BioTek, Winooski, VT, USA).

### 4.4. Total RNA Extraction and Full-Length cDNA Sequence Determination

The tentacles were rinsed several times with seawater and the tentacles and whole bodies of the jellyfish were used for total RNA extraction. The tentacles were ground in liquid nitrogen, and 1 g of the ground tentacle powder was dissolved in lysis buffer (200 mM Tris-HCl [pH 8.0], 0.7 M LiCl, 30 mM EDTA [pH 8.0], 7% SDS), and centrifuged at 13,000 rpm for 15 min at 4 °C. The supernatant was then transferred to a new microcentrifuge tube and the same volume of chloroform added. After vortexing, the tube was centrifuged under the same conditions and the step was repeated one more time. The supernatant was transferred to a new microcentrifuge tube, and 250 μL of propanol and 10 μL of glycerol (100%) were added. The tube was incubated at room temperature for 10 min and centrifuged at 12,000 rpm for 15 min at 4 °C. The pellet was washed with 70% ethanol and dried completely. It was then dissolved in diethyl pyrocarbonate (DEPC)-treated nuclease-free water and treated with DNaseI (NEB, Ipswich, MA, USA). The total RNA was finally heated to 75 °C for 10 min to inactivate the DNaseI and then used as the template for 5′ rapid amplification of cDNA ends (5′-RACE).

### 4.5. 3′- and 5′-RACE and cDNA Sequencing

3′-RACE of the *N. nomurai* CTRL-1 cDNA was performed with reverse transcription–PCR (RT–PCR) using a gene-specific forward primer (5′-GTGGTTGCCATGGAGATAGTGGTG-3′) and an oligo (dT)_18_ primer. The gene-specific primer was designed based on the partial nucleotide sequence of the *CTRL1* gene obtained from *N. nomurai* transcriptome sequencing data (Yum et al. [32], unpublished data). The RACE PCR for the *N. nomurai* jellyfish CTRL-1 cDNA was performed with the SMARTer RACE cDNA Amplification Kit (Clontech, Mountain View, CA, USA). In brief, the first-strand cDNA was synthesized from total RNA with a gene-specific reverse primer (5′-CACCACTATCTCCATGGCAACCAC-3′) and the SMARTer II oligonucleotide, according to the manufacturer’s instructions. PCR amplification was performed with the Advantage 2 PCR kit (Clontech, Mountain View, CA, USA) using a gene-specific reverse primer and a RACE long universal forward primer. The cycling parameters were: one cycle at 94 °C for 5 min; followed by 35 cycles at 94 °C for 30 s, 58 °C for 30 s, and 72 °C for 45 s; followed by one cycle at 72 °C for 10 min. Nested PCR amplification was performed under the same conditions, using a gene-specific reverse primer and a short universal primer. All the PCR products were purified with the Expin™ GeneAll® PCR SV purification kit (GeneAll, Seoul, Korea), cloned into the pGEM®-T Easy Vector System (Promega, Madison, WI, USA) and confirmed the clone by *EcoR*I digestion at 37 °C for 1 h. Full-length of cDNA sequence was identified by ABI PRISM 3739 Genetic Analyzer (Thermo Fisher, Waltham, MA, USA).

### 4.6. Genomic DNA Extraction and PCR

The genomic DNA was extracted from the whole bodies of *N. nomurai* jellyfish with the C-TAB method, after the whole bodies were grounded by liquid nitrogen. Briefly, 1 g of the ground powder was dissolved in 10 mL of lysis buffer (2% CTAB, 1.4 M NaCl, 100 mM Tris-Cl [pH 8.0], 20 mM EDTA [pH 8.0], 1% β-mercaptoethanol) and incubated at 65 °C for 1 h. After incubation, the genomic DNA was purified with 1 volume of phenol/chloroform/isoamyl alcohol (25:24:1; PCI) and treated with RNaseA (10 mg/mL). The supernatant was then purified with PCI and precipitated with the ethanol/sodium acetate (pH 5.2) method. The pellet was dissolved in DNase-free distilled water and used as the template for genomic DNA PCR. The specific primers for the amplification of *N. nomurai* *CTRL1* (forward: 5′-ATGTTGGCAATACTCATTCTTGGTC-3′; reverse: 5′-CTAGTATTTGATGTACTTGTTG-3′) were designed based on the full-length cDNA sequence. The cycling parameters were: one cycle at 94 °C for 5 min; followed by 35 cycles at 94 °C for 45 s, 58 °C for 45 s, 72 °C for 1 min; followed by a final extension at 72 °C for 10 min. The genomic DNA PCR product was purified, cloned and sequenced by the same method as above.

### 4.7. Phylogenetic and Nucleotide Sequence Analysis of CTRL1

The cDNA sequence of CTRL-1 from the *N. nomurai* jellyfish was subjected to a homology search with the NCBI BLAST program [33]. The CTRL-1 domains were predicted with the InterProScan search tool [34]. The signal peptide cleavage site in the deduced amino acid sequence was predicted with the SignalP 4.1 server [35]. The sequence identity values for the deduced amino acid sequence are available at the EMBL-EBI site [36]. A phylogenetic tree was constructed from the deduced amino acid sequence of *N. nomurai* CTRL-1 and those of other groups, based on the neighbor-joining method, a Poisson model, and uniform rates, with the MEGA ver. 6.06 software (Center for Evolutionary Medicine and Informatics, Arizona state University, Tempe, AZ, USA).

## Figures and Tables

**Table 1:** Comparison of *N. nomurai* CTRL-1 protein with those of other species with a BLAST analysis.

Class	Species	Identity (%)
Scyphozoa	*Aurelia aurita*	42
Hydrozoa	*Hydra vulgaris*	42
Actinopterygii	*Danio rerio*	48
	*Poecilia reticulate*	43
	*Stegastes partitus*	43
	*Salmon salar*	41
	*Takifugu rubripes*	44

## Supplementary Materials

The following are available online at www.mdpi.com/2072-6651/8/7/205/s1, Figure S1: Nemopilema nomurai chymotrypsin-like 1 (CTRL-1) genomic DNA sequence (GenBank accession No. KU_668697), composed of 2434 bp.
