# Supplementary material for: Cloning a Chymotrypsin-Like 1 (CTRL-1) Protease cDNA from the Jellyfish Nemopilema nomurai
Source: Toxins (Basel). 2016 Jul 5;8(7):205. doi: 10.3390/toxins8070205 (PMC4963838; doi:10.3390/toxins8070205)
Supplement: Supplementary file 1 [file toxins-08-00205-s001.pdf]

Supplementary Materials: Cloning a Chymotrypsin-Like 1 (CTRL-1) Protease cDNA from the Jellyfish Nemopilema nomurai

Yunwi Heo, Young Chul Kwon, Seong kyeong Bae, Duhyeon Hwang, Hye Ryeon Yang, Indu Choudhary, Hyunkyoung Lee, Seungshic Yum, Kyoungsoon Shin, Won Duk Yoon, Changkeun Kang and Euikyung Kim

ATGTTGGCAATACTCATTCTTGGTCTGTTCGTTGGCAGCAGTTTGGCCCAGCGTAAGCACCTTGGTTTAATTTGGCTTTGTTGAATAATCATTCAGAGA
AGCTGTTAAGTATATATTGCATTATATCTAAGTACCAATGAGAATAATACTAAGTCCATCTGTTGTTGATACTTGTCCTTTTCCAAGTGATACCCATTTACAGATTTTTGTCATCCTTGAGTTTCCCA
GATACTGATGACGGTCATTTTTTTGTCAGTTAAAAACTGATGACAGGTATTTGTTGTTGCAAATACAGCGACTTCTACCTTCCCCAAAATGGTGATCATAGATCTAGGATTATTGAGATGACATACA
AGCATTATATCTCAAGGTAGAAAGAAATAACTGCAGCGACCATTTATCATTAGCAAGTAGAGCAATGTAAGCTTCAAGGAACACCCAAACACAAAGCATTATTTCGCGTAATACGCAATATCATCA
TGGCTTCTTCTCGTGTATCCCGTGTCTTGCTGCTCAGTTCGACTAAACATTGTTCATTTACTAGAGCTGCTTGGTCTTAATATCAAATGCAGATTCTGAATACCTCCTAAATATATCACACGCTTCT
TGCAG**AATGTGGCGTTCCCCGATACGCGGCTAGTCGGGTCATTGGTGGCAGCACTGCGAGGCCTGGTTCCTGGCCTTGGCAA**
GTAGCCATCTACTACGACAACCGTTTTCATTGTGGAGGATCGTTGGTGAACGCCAACTGGGTTGTTACTGCTGCTCATTGCC
TTGATAGAACCAGGATGAGCGGATTCACCATTGTTTTGGGTGAGTTGGACTAATTTGCAATCATGTCATGGCTTCATATTAGATATCCAAGCAACGATGAGTCTTT
AGTCTCAGCAAACTCCAGTGGCTATTGGTTAAGGAACTTTAGTTTGTGATCTTCTATCATAATTCCAAGTTGGTATAGACTCAGCAGTGAGATTCTAATTTATGATCATTCCCTTTCTTAAGATAGA
ACTGGCGGTCATCCAGTGAGAAATAAACTTGTTTGTTATGCAACATTAATGCACTGTAATAAGACAATGGTTTGAATTTCAATGTATTGTTATTGCAAATACCATGTATTTGCTAATTAGATGGGGG
TACCACTGAGTACTATAGATAAGGTTAGAATACCAAGGTCTGCACTTGATACACACGACAGGACTTGCTTTGGACGAACATTGGAGACAGGAAAGAGGGAGAATGGGGGTCATTAAAGCAAATG
TGAAGTACAGAAGAGAGAACTTTGTTCAATAAAAGATAGTAGAAAGAAAAATATAAATGAGAGCATTGTATAGTATTGACTTCATTTCTCTTTAATAGCTAACATGTACCAACCCCTTTCAAATCCA
ACGAAATTGCTTTAAAATCTTTTAAAGAATATGATTTTCACTCAAAAATTGGGCTTCCTTATACTATACTACCTACAAATACTGTTCTTTTTTCTCGAGTCCCTTCTTGTGAAAATTTCAATTATTGAC
GAGATGATGTTATCTCTTTTAAG**GTGAGCACTACCGACAACAAAAGGAGGGAAGCGAGCAATATTTCAAAGCCAAGAGGGCATTCACG**
CATCCTCAGTACAACAAACAGCCGCTGGACAGCGACATCGCGCTCATCAAGCTGGATAGGCCAGCTCAGTTCAACAACCGA
GTGCAGCCAGTCTGTCTGCCATCCACCTACAACAAGCCCCTTCCCGGCACCACCTGCTTCATCACTGGTAAGGCGTTGCCAGATCCACG
CTTTTACTCGAGAAGCGTATTTGAAAGAATTAAGTGTCCTCTGAGACAATAAACAATGACACTAGTTGAAATCCATGGGGCTTATGGTTATCCTAAAAATGAGCATTAATCTTGTTTGGAGTCTAG
TGGCGATGTAGACCAGACTCAAGGGAGATTTGTCTCTCTATCCTTATATGAAAATTTGGTACTGTTGCTTATCACTGTTAGAGATAACAAGGAAATATGCCTCTCCTTTTAGCGTTCTTAAAGGAG
TCAGAAGAAAAAAATTCCTGCTGGCAATCATTGAATACTTCTAGCATTTAAGCAATGGATTACTTCTGTATTCTAG**GTTGGGGAAAGACATCACATCCCGGGTCCTCAG**
CTTACGTTCTCCAACAATCACCACTCCCGGTCGTTGACAACAGAAGATGCCACGCCCTGAACAAACCAAACACTCGAATCG
GCATCGCAGGCAACATGCTCTGCGCTGGTTTCGGGCCTAACGACATCCGAAGTGGTTGCCATGGAGATAGTGGTGGCCCAT
TCGTATGCAAAAGTGGCAGCCAATGGTCCCTCCAGGGAGCAGTGAGCTGGGGCTCCGGACGTTGCAACACGCGAGATGCA
TACACTGTATTCGCAAGGGTCACAAACTTCGTCAGTTGGATCAACAAGTACATCAAATACTAG

Figure S1. Nemopilema nomurai chymotrypsin-like 1 (CTRL-1) genomic DNA sequence (GenBank accession No. KU_668697), composed of 2434 bp.
